# Influence of genetic polymorphisms on gefitinib pharmacokinetics and adverse drug reactions in non-small cell lung cancer patients

**DOI:** 10.1007/s10555-025-10299-7

**Published:** 2025-11-06

**Authors:** Prathvi V. Shenoy, Gayathri Baburaj, Rama Rao Damerla, Ananth Pai, Sharada Mailankody, Murali Munisamy, Surulivelrajan Mallayasamy, Karthik S. Udupa, Jill Kolesar, Mahadev Rao

**Affiliations:** 1 Department of Pharmacy Practice, Manipal College of Pharmaceutical Sciences, Manipal Academy of Higher Education, Manipal, 576104 Karnataka India; 2 Department of Medical Genetics, Kasturba Medical College Manipal, Manipal Academy of Higher Education, Manipal, 576104 Karnataka India; 3 Department of Medical Oncology, Kasturba Medical College Manipal, Manipal Comprehensive Cancer Care Centre, Manipal Academy of Higher Education, Manipal, 576104 Karnataka India; 4 Department of Translational Medicine, All India Institute of Medical Sciences, Bhopal, 462020 Madhya Pradesh India; 5 Department of Pharmaceutical Sciences and Experimental Therapeutics, College of Pharmacy, University of Iowa, Iowa City, IA USA

**Keywords:** Gefitinib, Non-small cell lung cancer, Pharmacokinetics, Single nucleotide polymorphisms, Adverse drug reactions

## Abstract

Gefitinib is a first-generation epidermal growth factor receptor (EGFR) tyrosine kinase inhibitor (TKI) used in the treatment of non-small cell lung cancer (NSCLC). It is metabolized extensively in the liver by enzymes encoded by *CYP3A4*, *CYP2D6,* and *CYP3A5* and is the substrate of membrane transporters including *ABCB1* and *ABCG2*. Evidence shows that single-nucleotide polymorphisms (SNPs) in these metabolizing and transporting genes contribute to the inter-individual variability in gefitinib response and development of gefitinib-induced adverse drug reactions (ADRs). This narrative review identifies the existing literature exploring the impact of SNPs on plasma gefitinib concentration and ADRs in patients with NSCLC receiving gefitinib therapy. SNPs were identified in drug-metabolizing enzyme coding genes, including *CYP3A4, CYP2D6, CYP3A5,* and other CYP homologs, drug transporters including *ABCB1, ABCG2, SLCO1B1* and other genes, including *UGT1A7* and *FOXO3.* Current research has not identified any genetic association between specific SNPs in *ABCB1, OATP1B1,* and *UGT1A7* and the pharmacokinetics of gefitinib. Additionally, most of these studies focused on individual SNP associations; however, it may be more important to consider them in combination to better understand their collective impact on gefitinib ADR. Hence, further comprehensive research is essential to examine these genetic variants across different ethnic groups, monitor the drug-drug interactions, and study the phenoconversion to draw definitive conclusions about the pharmacokinetics of gefitinib. This could lead to the development and implementation of a genotyping-based approach for gefitinib dosage optimization in clinical settings.

**Supplementary Information:**

The online version contains supplementary material available at 10.1007/s10555-025-10299-7.

## Introduction

According to GLOBOCAN 2022, lung cancer is the most frequently diagnosed cancer. It accounts for 12.4% of all cancers and is the leading cause of cancer-related mortality worldwide, with an estimated 18.7% of deaths [1]. Non-small cell lung cancer (NSCLC) is the predominant type of lung cancer, comprising approximately 85% of the total cancer cases [2]. Treatment options for NSCLC vary depending on the stage of the disease. Patients with early-stage NSCLC resectable tumors may be cured either by surgery alone or by combining surgery with chemotherapy. For patients with advanced-stage NSCLC with unresectable tumors, treatment is often biomarker-directed and may include targeted therapies, immunotherapy, and/or chemotherapy, but a cure is rare in this setting [3].

In more than half of patients with NSCLC, the signaling pathway of the epidermal growth factor receptor (EGFR) is activated and is the cornerstone for oncogenesis. Activation of EGFR signaling could be due to protein overexpression, gene copy number variation (CNV), or genetic mutations in *EGFR* [4]. Approximately 10–15% of the NSCLC diagnoses are due to these *EGFR* driver genetic mutations. In India, the prevalence of *EGFR* mutation rates is higher (30%) than any other genomic mutations in lung cancer patients [5]. In such cases, EGFR tyrosine kinase inhibitors (TKIs) are the first-line therapy to treat NSCLC patients. EGFR-TKIs have demonstrated superior efficacy compared to traditional platinum-based chemotherapy [6]. Gefitinib, a first-generation TKI, has shown improved survival outcomes, higher response rates, and significantly longer progression-free survival (PFS) relative to standard first-line chemotherapy in patients with advanced NSCLC harboring activating mutations of the *EGFR* gene and has been linked to enhanced tolerability and quality of life compared to chemotherapy [7–10]. NSCLC patients with *EGFR* somatic mutations, such as exon 19 deletions, nucleotide substitutions including Gly719X (G719X) in exon 18, Ser768Ile (S768I) in exon 20, Leu861Gln (L861Q), and Leu858Arg (L858R) missense mutations in exon 21, are therapeutically treated with an EGFR-TKI inhibitor, gefitinib [7, 8, 11]. Overall/Objective response rates (ORRs) among patients with EGFR mutation-positive cancer cells receiving gefitinib have been documented to range from 62 to 85% [7–10]. Subsequent head-to-head trials, comparing EGFR-TKIs, revealed that second-generation TKIs, such as afatinib and dacomitinib, provided longer PFS than the first-generation TKI, gefitinib [12, 13]. However, it was observed that second-generation TKIs were associated with a higher rate of adverse events compared to gefitinib [12–15].

Although patients initially respond well to gefitinib therapy, resistance to treatment eventually develops. Multiple mechanisms underlying acquired resistance have been identified, which can be categorized into secondary *EGFR* mutations, activation of alternative signaling pathways, and phenotypic or histological transformations [16–18]. Among these, the most prevalent mechanism is the Thr790Met (T790M) mutation, responsible for 50–60% secondary resistance cases to first-line EGFR TKI therapy [19]. This is one of the main reasons for the development of the third-generation EGFR TKIs, such as osimertinib, which is more effective than all other TKIs [20]. However, a study has shown that PFS and overall survival (OS) were significantly prolonged when pemetrexed and carboplatin chemotherapy were added to gefitinib, exhibiting effects comparable to those with osimertinib [21]. Therefore, owing to its cost-effectiveness and demonstrated clinical activity, gefitinib remains an acceptable treatment option, offering acceptable clinical outcomes at a lower cost [22–24].

Gefitinib (Iressa™, ZD1839) is a first-generation EGFR-TKI, which was approved for clinical use by the U.S. FDA (United States Food and Drug Administration) in 2003 [25]. It is an orally administered drug that is slowly absorbed, with an average bioavailability of 60%. The recommended dosage of gefitinib is 250 mg administered once daily, with no routine dose adjustment required [11, 26]. It reaches peak plasma levels, T_max_, within 3 to 7 h post-administration, and the mean half-life (t_1/2_) for a single dose is approximately 41 h [26, 27]. Steady-state plasma concentration is achieved within 10 days, and the steady-state apparent volume of distribution is 1400 L, suggesting that the drug is extensively distributed into the body tissues [26, 27]. Gefitinib has a plasma protein affinity of 90% and binds to human serum albumin and α−1-acid glycoprotein (AGP) [28].

**Fig. 1:**
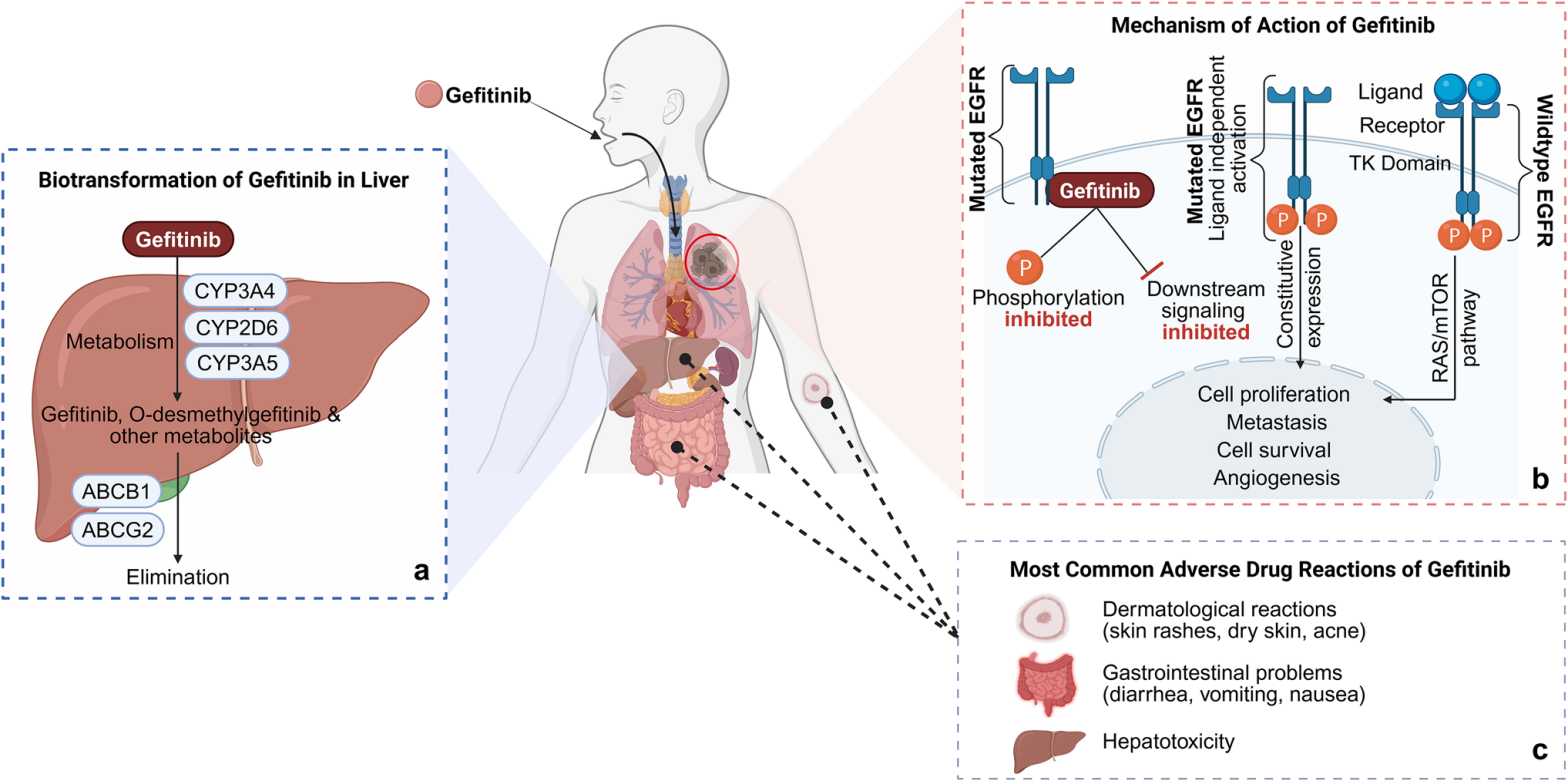
Schematic diagram showing: (**a**) Biotransformation of gefitinib in the liver, (**b**) Mechanism of action of gefitinib, and (**c**) Adverse drug reactions of gefitinib. Created with BioRender.com (Agreement number: YG28UME5C0). **Footnote:** EGFR: Epidermal growth factor receptor, TK: Tyrosine kinase, RAS/mTOR: Rat sarcoma virus/mammalian target of rapamycin, CYP3A4: Cytochrome P450 family 3 subfamily A member 4, CYP2D6: Cytochrome P450 family 2 subfamily D member 6, CYP3A5: Cytochrome P450 family 3 subfamily A member 5, ABCB1: Adenosine triphosphate binding cassette subfamily B member 1, ABCG2: Adenosine triphosphate binding cassette subfamily G member 2

The primary site for gefitinib metabolism is the liver, where it undergoes extensive biotransformation (Fig. 1a). It is metabolized by the cytochrome P450 (CYP450) family of enzymes, primarily via CYP3A4, while the other CYPs, including CYP2D6 and CYP3A5, play a minor role [29]. The major active metabolite of gefitinib in human plasma is O-desmethyl-gefitinib (M523595), which is formed by CYP2D6 and circulates at concentrations like of the parent compound [29]. Additionally, other minor metabolites (including M537194, M295820, M387783, M527301, M594557, M605211, and M605212) have also been identified in human plasma at significantly lower levels [26, 30]. A study by McKillop *et al*., also reported several gefitinib metabolites from human liver microsomes *in vitro* [31]*.* Currently, over 40 gefitinib metabolites, intermediates and glutathione adducts have been identified [32]. The plasma clearance of gefitinib is around 500 mL/min and is predominantly excreted in the feces, with less than 7% in the urine.

Gefitinib is a competitive, reversible inhibitor of the tyrosine kinase domain of the EGFR, which disrupts the signaling in cancer cells with mutant or overactive EGFR [27]. It competes with adenosine triphosphate (ATP) for tyrosine kinase binding sites on the EGFR, inhibiting EGFR autophosphorylation and downstream signaling (Fig. 1b). This inhibition leads to the suppression of cancer cell growth [29].

There are substantial inter-individual differences in the clinical outcomes of gefitinib, possibly due to single-nucleotide polymorphisms (SNPs) in genes responsible for metabolism and transport [33]. Therefore, identifying biomarkers that accurately predict clinical response to this medication is crucial for optimizing treatment outcomes and minimizing adverse effects, thereby facilitating personalized therapy [8, 34]. Several studies have focused on the impact of the pharmacogenomics of gefitinib on therapy outcomes, where the ADME (absorption, distribution, metabolism, and excretion) processes of gefitinib were determined by identifying gene polymorphisms in enzymes. Genetic variation in these pharmacokinetics and pharmacogenomics-related enzymes significantly impacts the effectiveness of gefitinib [4, 35]. Therefore, the purpose of this review was to provide a comprehensive overview of the SNPs within genes encoding the drug-metabolizing enzymes and transporters implicated in gefitinib pharmacokinetics. Additionally, we analyzed the allele frequencies of SNPs implicated in the pharmacokinetics of gefitinib, available in the *“All of Us”* research program database. Such knowledge is essential for understanding interindividual variability in drug response, potential drug interaction, and the development of resistance [36].

**Fig. 2:**
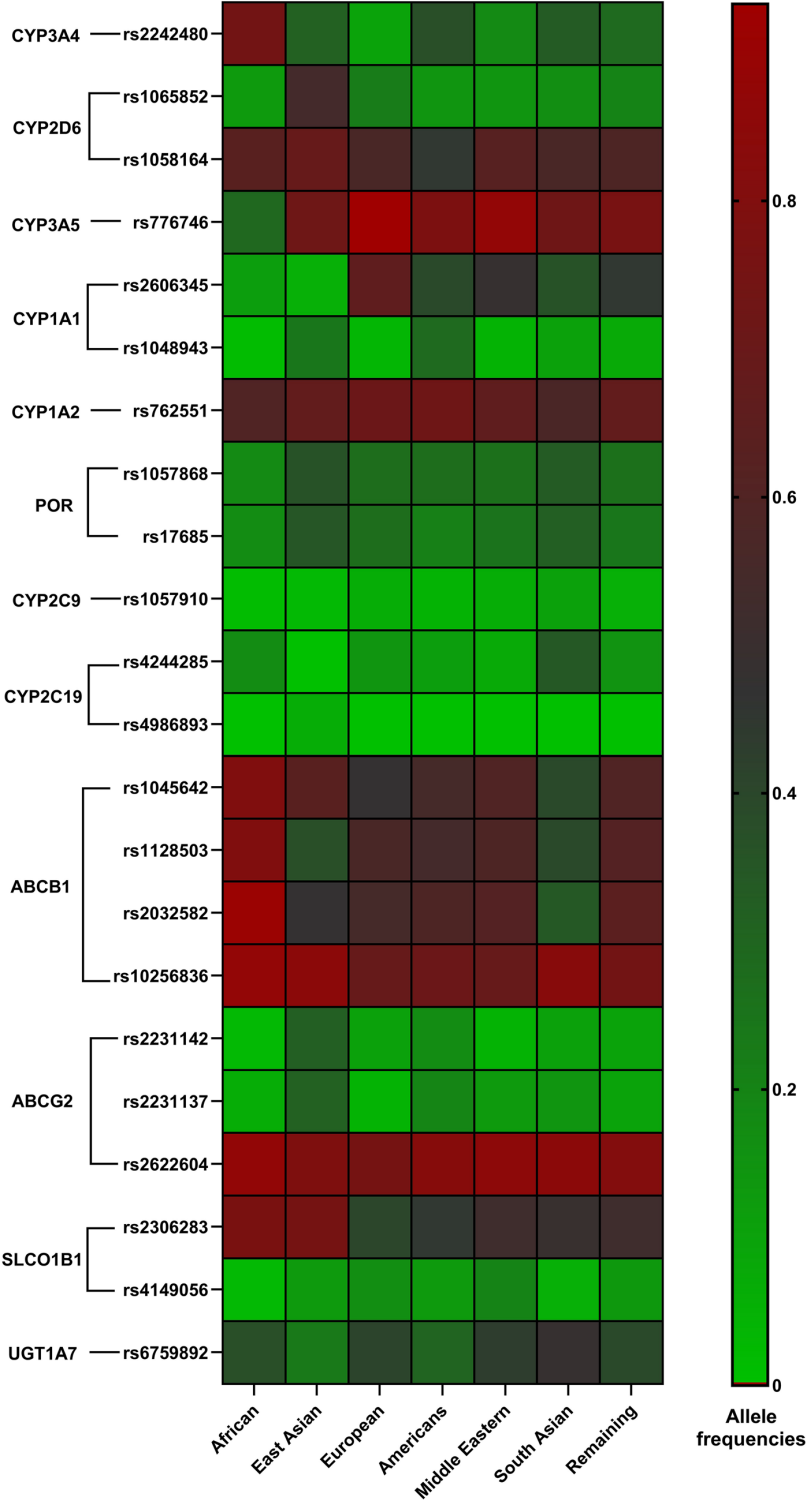
Heatmap showing the allele frequencies of the SNPs described in the manuscript based on the different ethnic populations as described in the public tier from the “*All of Us”* Research Program database. Graph created using GraphPad Prism (version 10.4.1). **Footnote:** *CYP3A4*: Cytochrome P450 family 3 subfamily A member 4, *CYP2D6*: Cytochrome P450 family 2 subfamily D member 6, *CYP3A5*: Cytochrome P450 family 3 subfamily A member 5, *CYP1A1*: Cytochrome P450 family 1 subfamily A member 1, *CYP1A2*: Cytochrome P450 family 1 subfamily A member 2, *POR*: Cytochrome P450 oxidoreductase, *CYP2C9*: Cytochrome P450 family 2 subfamily C member 9, *CYP2C19*: Cytochrome P450 family 2 subfamily C member 19, *ABCB1*: Adenosine triphosphate binding cassette subfamily B member 1, *ABCG2*: Adenosine triphosphate binding cassette subfamily G member 2, *SLCO1B1*: Solute carrier organic anion transporter family member 1B1, *UGT1A7*: UDP glucuronosyltransferase family 1 member A7

The following Medical Subject Headings (MeSH) terms were used as part of the literature search: gefitinib, non-small cell lung cancer, genetic polymorphism, genetic susceptibility, pharmacogenomics, single-nucleotide polymorphisms, and pharmacokinetics. We focused on studies evaluating the association between genetic variants and gefitinib pharmacokinetic outcomes in patients with NSCLC, regardless of age, receiving gefitinib therapy. We excluded all the non-English language studies and those that did not formally evaluate the effects of genotype on gefitinib exposure. Additional relevant literature was identified through cross-referencing. Information about the SNPs and their corresponding genes was catalogued using various online databases such as Ensembl, Online Mendelian Inheritance in Man (OMIM), GeneCards, National Center for Biotechnology Information (NCBI) dbSNP, Pharmacogene Variation (PharmVar), VariantValidator, Mutalyzer 3, and MutationTaster (Table S1) [37–44]. Additionally, the public tier data from the “*All of Us”* Research Program database was used to record the allele frequencies of the SNPs influencing the pharmacokinetics of gefitinib across various ethnic populations (Fig. 2) [36]. The heatmap reveals distinct patterns of allele frequency across populations, highlighting population-specific genetic differences. For instance, rs2242480 (*CYP3A4)* exhibits a high allelic frequency in African populations (0.74) but is markedly lower in Europeans (0.09). Similarly, multiple SNPs in the *ABCB1* gene, including rs1045642, rs1128503, and rs2032582, display elevated frequencies in African populations compared to other groups. Conversely, certain variants exhibit notably low frequencies or are nearly absent from specific populations. For instance, rs776746 (*CYP3A5)* is prevalent across most ethnic groups but has a comparatively low frequency in African populations (0.29). Additionally, rs4986893 (*CYP2C19)* is absent in all populations except East Asians, where it is present at a low frequency (0.06), and is consistent with the literature [45]. Similarly, rs1057910 (*CYP2C9*) demonstrates minimal frequencies across most populations. Beyond these population-specific differences, some genetic variants show consistently high frequencies across diverse populations. Notably, rs10256836 (*ABCB1*)*,* rs2622604 (*ABCG2*)*,* and rs762551 (*CYP1A2*) all exhibit high frequencies (> 0.5) across all populations.

Clinical investigations have evaluated ethnic variations in the pharmacokinetics of gefitinib, showing no clinically significant differences in systemic exposure among ethnic groups [26]. Population pharmacokinetic studies involved patients from various ethnicities, primarily Asian and Caucasian groups, because of the high prevalence of *EGFR* mutations in East Asian individuals with NSCLC, enabling significantly superior survival outcomes [46]. The analysis of allele frequencies in candidate gene variants and SNPs holds significant implications for various scientific domains, including uncovering the genetic correlations with diseases and health-related traits, and estimating the prevalence of individuals predisposed to certain conditions or drug resistance within populations [47].

## Association of SNPs on gefitinib pharmacokinetics

### Drug-metabolizing enzymes

#### Cytochrome P450 family 3 subfamily A member 4 (CYP3A4)

*CYP3A4* is a protein-coding gene with a reverse orientation, composed of 13 exons (transcript: NM_017460.6 at chromosome 7q22.1: 99,756,967–99,784,184) and codes for a 503 amino acid long protein. The CYP3A4 enzyme catalyzes the oxidative metabolism of approximately 60% of the clinically used drugs. Studies have revealed that alterations in the enzymatic activity or expression levels of *CYP3A4* are key determinants of drug efficacy and toxicity [48]. Over 10,000 genomic variants have been identified in the *CYP3A4* gene [49]. Among these, rs2242480 has been commonly recognized and well-studied [50].

The rs2242480 (NM_017460.6: c.1026 + 12G > A) is a SNP found within intron 10 of *CYP3A4*. The allele frequencies reported by the 1000 Genome Project are C = 0.5783 and T = 0.4217, indicating C to be the major allele and T as the minor allele [51]. Studies have demonstrated the influence of intronic variants on their corresponding enzyme activity, but the molecular mechanism underlying their effect on drug metabolism in the liver is still unknown [52, 53]. A study by He *et al.,* emphasized that rs2242480 increased the metabolic activity of the CYP3A4 enzyme, thereby resulting in increased metabolic clearance of the drug [54]. In line with these findings, Ma Y *et al.,* demonstrated a statistically significant association between rs2242480 with gefitinib trough concentration (C_trough_), where the overall interindividual variability was substantial ranging from 47.8 to 470 ng/mL (median 175 ng/mL), where patients carrying the CC + CT genotype had lower C_trough_ when compared to those with the TT genotype (*p* = 0.019), suggesting the influence of polymorphism on gefitinib pharmacokinetics [55]. Chen *et al.,* reported higher AUC (area under the curve) of gefitinib in individuals who were carriers of the CC genotype (AUC_0-t_ = 6157.34 ± 2049.21 ng.h/mL and AUC_0-∞_ = 6624.62 ± 2227.05 ng.h/mL) than in those with CT/TT genotype (AUC_0-t_ = 5051.26 ± 1575.83 ng.h/mL and AUC_0-∞_ = 5361.12 ± 1807.07 ng.h/mL) (unadjusted *p* < 0.05), indicative of a lower clearance of the drug in those with CC genotype, in healthy subjects [56]. While certain studies reported an association between rs2242480 with gefitinib pharmacokinetics, these findings could not be consistently reproduced in others, as there were no appreciable changes in the pharmacokinetic parameters across genotype groups and were also not statistically significant (Table 1). These discrepancies could be due to various factors such as variations in the sample size, ethnic differences, disparities in sample collection methods, or analytical inconsistencies. Further research is essential to examine the functional impact of rs2242480 polymorphism on gefitinib pharmacokinetics in different ethnic populations.

**Table 1:** Influence of rs2242480, a *CYP3A4* genetic variant, on pharmacokinetic (PK) parameters and efficacy of gefitinib

Authors	Population	Sample size	Criteria for sample collection	Time of sample collection	PK parameters	Efficacy
Kobayashi, *et al*., 2015 [35]	Japan	31 NSCLC patients	14 days after gefitinib therapy (250 mg OD)	Predose, 1, 2, 4, 6, 8, 12, and 24 h post-dose	AUC_0–24_, C_trough_ – non -significant	NR
Hirose *et al*., 2016 [57]	Japan	35 NSCLC patients	Between day 1 to day 15 of gefitinib therapy (250 mg OD)	Day 1: Predose and 1,3, 5, 8, and 24 h post-dose Day 8: Pre-dose Day 15: Pre-dose	AUC_0–24_, C_max_ – non-significant	PFS – non-significant
Ma *et al*., 2019 [55]	Chinese	58 NSCLC patients	Gefitinib therapy – 250 mg OD 1 st occasion: Before gefitinib dosage 2nd occasion: Cycle 2^**$**^	0 h (baseline—before first gefitinib dose) and 5 min before gefitinib administration at day 1 of cycle 2^**$**^	Lower gefitinib C_trough_ in patients with CC/CT genotypes than patients with TT genotype	PFS and ORR: non-significant
Wan *et al*., 2020 [58]	Chinese	39 healthy volunteers	Single oral dose of 250 mg of gefitinib	Predose and at 0.5, 1, 2, 3, 4, 5, 6, 7, 8, 10, 12, 24, 48, 72, 96, 120, 144 and 168 h post-dose	C_max_, T_max_, t_1/2_, AUC_(0–168 h)_, AUC_(0-∞)_, CL/F – non-significant	NR
Chen *et al*., 2024 [56]	Chinese	45 healthy male volunteers	16 days after gefitinib therapy (250 mg OD)	0 h (pre-dose), 0.5, 1, 1.5, 2, 2.5, 3, 3.5, 4, 4.5, 5, 5.5, 6, 6.5, 7, 8, 12, 24, 48, 72, 96, 120 and 144 h post-dose	Higher gefitinib AUC_0-t_ and AUC_0-∞_ in CC genotype than in CT/TT genotype	NR

^**$**^One month was counted for one cycle. Non-significant: refers to instances where the observed changes in PK parameters and efficacy did not reach statistical significance in the cited studies. NR (Not reported): denotes that the cited study did not evaluate or provide data on efficacy. Abbreviations: NSCLC: Non-small cell lung cancer, OD: Once daily, C_trough_: Trough concentration, AUC: Area under the curve, C_max_: Maximum concentration, T_max_: Time of C_max_, t_1/2_: Half-life of drug, CL/F: Oral plasma clearance, PFS: Progression-free survival, ORR: Objective response rate

Although rs2242480 has been associated with altered C_trough_ or AUC in a few studies, the magnitude of these observed changes remains modest and falls within the broad inter-patient variability already reported by the FDA for gefitinib pharmacokinetics [26]. In the study by Ma Y *et al.,* despite a statistically significant difference in the plasma concentration, the gefitinib C_trough_ values were not correlated with ORR or PFS, indicating that the exposure differences attributed to this variant did not translate into clinically meaningful treatment outcomes [55]. Similarly, Chen *et al.,* observed that while CC genotype carriers exhibited higher AUC compared to CT/TT carriers, these associations did not remain statistically significant after applying the Benjamini–Hochberg correction for multiple testing (BH-adjusted p-values of 0.165 and 0.296 for AUC_0-t_ and AUC_0-∞_ respectively). Moreover, this modest increase in exposure was not associated with a higher incidence of adverse drug reactions (ADRs), despite a trend towards higher ADRs in CC carriers. Collectively, these findings indicate that the pharmacokinetic differences associated with rs2242480, though statistically suggestive in single-variant analyses, contribute only marginally to the overall variability in gefitinib exposure and fall within a range unlikely to necessitate clinical dose modification. This interpretation is further supported by evidence that even larger differences in exposure – such as the three-fold increase observed in patients with hepatic impairment – have not warranted dose adjustments in clinical practice [26].

#### Cytochrome P450 family 2 subfamily D member 6 (CYP2D6)

*CYP2D6* is an essential pharmacogene with established pharmacogenetic associations implemented in clinical practice for the therapeutic management recommended by the U.S. FDA and Mayo Clinic Laboratories [59, 60]. This gene comprises 9 exons (transcript: NM_000106.6 at chromosome 22q13.2: 42,125,531–42,130,881) and codes for a protein with 497 amino acids. With over 4000 reported genetic variants, *CYP2D6* is one of the most polymorphic genes [61]. The CYP2D6 enzyme metabolizes over 25% of the clinically used drugs and is among the most extensively studied CYP genes in the context of phenoconversion [62]. Over 135 distinct star (*) alleles of *CYP2D6* have been characterized and catalogued by the PharmVar Consortium. The metabolic activity of the CYP2D6 enzyme is classified into four groups: ultra-rapid metabolizers (UM), extensive metabolizers (EM), intermediate metabolizers (IM), and poor metabolizers (PM). UM and EM exhibit normal or enhanced functions, while IM and PM have reduced activity. These alleles are classified according to their enzyme function as normal, decreased, absent, or unknown, as defined by the Clinical Pharmacogenomics Implementation Consortium (CPIC) guidelines [63]. Alleles like CYP2D6*1 and *2 have normal functions, while CYP2D6*10, *14, *29 and *41 have decreased or impaired functions and CYP2D6*4 and *5 have no function [64]*.* Individuals with multiple copies of functional *CYP2D6* alleles are classified as UM. EM includes a combination of one or two functional alleles, IM includes two impaired alleles, and PM includes two non-functional alleles [65]. One of the pioneer studies by Swaisland *et al.,* conducted on healthy volunteers, reported that AUC_∞_ was significantly higher in PM individuals when compared to EMs (3060 vs 1430 ng.h/mL; *p* < 0.05) [66]. They also reported a higher C_max_ in PMs than EMs (93.0 vs 61.5 ng/mL) [66]. A recent study by Nio *et al.,* revealed that AUC_0–48_ of gefitinib was higher (12.6 ± 2.2 μM h) and AUC_0–48_ of O-desmethyl gefitinib was lower (3.23 ± 2.2 μM h) in patients with CYP2D6*5/*10 or *10/*10 genotype, while patients with CYP2D6*1/*1 genotype had lower AUC_0–48_ of gefitinib (5.52 ± 0.61 μM h) and higher AUC_0–48_ of O-desmethyl gefitinib (28.2 ± 23 μM h) [67]. On the contrary, Kobayashi *et al.,* found no significant alteration in AUC_0–24_ of gefitinib and *CYP2D6* genotypes (*p* = 0.467). However, the study could find a significant association between the AUC_0–24_ of O-desmethyl gefitinib and *CYP2D6* genotype, where the AUC_0–24_ was lower (1460 ng.h/mL) in IMs of *CYP2D6* and was higher (12,523 ng.h/mL) in EMs of *CYP2D6* (*p* = 0.021) [68]. No other studies were able to infer any association between *CYP2D6* polymorphisms and gefitinib pharmacokinetics (Table 2).

**Table 2:** Influence of *CYP2D6* genetic variants and star alleles on PK parameters and efficacy of gefitinib

Authors	Population	Sample size	Criteria for sample collection	Time of sample collection	Star alleles/SNP ID	PK parameters	Efficacy
Swaisland *et al*., 2006 [66]	Single centre in the UK and Germany	73 healthy volunteers	Single oral dose of gefitinib (50–500 mg)	Scheduled time points for up to 10 days post dose	*CYP2D6* PMs versus EMs	AUC_∞_, C_max_ are higher in PMs compared to EMs	NR
Kobayashi, *et al*., 2015 [35]	Japan	31 NSCLC patients	14 days after gefitinib therapy (250 mg OD)	Predose, 1, 2, 4, 6, 8, 12, and 24 h post-dose	CYP2D6*5 (Full gene deletion)	AUC_0–24_, C_trough_ – non-significant	NR
					CYP2D6*10 (rs1065852)	AUC_0–24_, C_trough_ – non-significant	NR
Hirose *et al*., 2016 [57]	Japan	35 NSCLC patients	Between day 1 to day 15 of gefitinib therapy (250 mg OD)	Day 1: Predose and 1,3, 5, 8, and 24 h post-dose Day 8: Pre-dose Day 15: Pre-dose	CYP2D6*10 (rs1065852)	AUC_0–24_, C_max_ –non-significant	PFS: non-significant
Kobayashi, *et al*., 2016 [68]	Japan	36 NSCLC patients	Day 14 of 250 mg OD gefitinib therapy	Predose and at 1, 2, 4, 6, 8, 12, and 24 h post-dose	*CYP2D6* EMs versus IMs	AUC_0–24_ – non-significant	NR
Wan *et al*., 2020 [58]	Chinese	39 healthy volunteers	Single oral dose of 250 mg of gefitinib	Predose and at 0.5, 1, 2, 3, 4, 5, 6, 7, 8, 10, 12, 24, 48, 72, 96, 120, 144 and 168 h post-dose	CYP2D6*10 (rs1065852)	C_max_, T_max_, t_1/2_, AUC_(0–168 h)_, AUC_(0-∞)_, CL/F – non-significant	NR
					CYP2D6*29 rs1058164	C_max_, T_max_, t_1/2_, AUC_(0–168 h)_, AUC_(0-∞)_, CL/F – non-significant	NR
Nio *et al*., 2022 [67]	Japan	18 NSCLC patients who are 75 years or older	Day 1 of gefitinib therapy (250 mg OD)	Predose, 1, 2, 4, 6, 8, 24, and 48 h post-dose	CYP2D6*5 (Full gene deletion) and CYP2D6*10 (rs1065852)	Highest gefitinib AUC_0–48_ in patients with CYP2D6*5/*10 or *10/*10 genotype	NR
Chen *et al*., 2024 [56]	Chinese	45 healthy male volunteers	16 days after gefitinib therapy (250 mg OD)	0 h (pre-dose), 0.5, 1, 1.5, 2, 2.5, 3, 3.5, 4, 4.5, 5, 5.5, 6, 6.5, 7, 8, 12, 24, 48, 72, 96, 120 and 144 h post-dose	CYP2D6*29 rs1058164	T_max_, t_1/2_, C_max_, AUC_0-t_, AUC_0–1_, Vd, CL/F – non-significant	NR

Non-significant: refers to instances where the observed changes in PK parameters and efficacy did not reach statistical significance in the cited studies. NR (Not reported): denotes that the cited study did not evaluate or provide data on efficacy. Abbreviations: NSCLC: Non-small cell lung cancer, *CYP2D6*: Cytochrome P450 family 2 subfamily D member 6, OD: Once daily, AUC: Area under the curve, C_trough_: Trough concentration, C_max_: Maximum concentration, T_max_: Time of C_max_, t_1/2_: Half-life of drug, Vd: Volume of Distribution, CL/F: Oral plasma clearance, PFS: Progression-free survival

*CYP2D6* PMs demonstrated approximately two-fold higher gefitinib exposure compared to EMs [26], as was consistently replicated by Swaisland *et al.,* and Nio *et al.,* [66, 67]. While this magnitude of change contributes to increased risk of ADRs, the broad overlap in exposure between *CYP2D6* phenotypes and other variability sources complicates its clinical interpretation [26]. Gefitinib’s significant inter-individual pharmacokinetic variability leads to overlapping exposure ranges across various *CYP2D6* phenotypes, rendering genotype-guided dose modifications unnecessary [26]. Additionally, PharmGKB labels *CYP2D6* as “actionable PGx”, suggesting dose adjustments, contraindication or alternate drug recommendation, or other guidance for patients with a particular genotype or metabolizer phenotype, if known. However, the label does not require or recommend genotype or phenotype testing before using the drug [61]. Collectively, these findings indicate that while *CYP2D6* polymorphism contributes to gefitinib’s pharmacokinetic variability, the magnitude of its effect falls within the drug’s established therapeutic range and does not necessitate routine pharmacogenetic testing in clinical practice.

#### Cytochrome P450 family 3 subfamily A member 5 (CYP3A5)

CYP3A5 is another CYP enzyme that plays a minor role in the metabolism of gefitinib. It is encoded by *CYP3A5* gene, which comprises 13 exons (transcript: NM_000777.5 at chromosome 7q22.1: 99,648,194—99,679,996) and encodes a 502 amino acid long protein. Over 11,000 genetic variants have been identified and listed in the NCBI dbSNP database, highlighting the polymorphic nature of this gene [69]. Over 9 distinct star (*) alleles of *CYP3A5* have been characterized and cataloged by the PharmVar Consortium [70]. Of these, CYP3A5*3 allele is well characterized. It corresponds to rs776746 (NM_000777.5: c.219-237A > G), which is an intronic variant present in intron 3 of the *CYP3A5* gene. It is a putative splice site disruptor, as evidenced by its association with aberrant splicing and predictions from the Human Splicing Finder (HSF) database [71, 72]. Chen *et al.,* in their study with healthy volunteers, found a higher gefitinib AUC_0-t_ and AUC_0-∞_ in the carriers of CC genotype (AUC_0-t_ = 6303.03 ± 2132.14 ng.h/mL; AUC_0-∞_ = 6784.77 ± 2368.43 ng.h/mL) than in CT/TT genotype (AUC_0-t_ = 4954.13 ± 1417.35 ng.h/mL; AUC_0-∞_ = 5254.35 ± 1590.89 ng.h/mL) (*p* < 0.05) [56]. This was the only study to establish an association between CYP3A5*3 and gefitinib concentration. Previous research has not identified any such association (Table S2).

#### Other CYPs

In addition to the aforementioned CYPs, studies have also delved into other CYP homologs, including cytochrome P450 family 1 subfamily A member 1 (*CYP1A1*), cytochrome P450 family 1 subfamily A member 2 (*CYP1A2*), cytochrome P450 family 2 subfamily C member 9 (*CYP2C9*), cytochrome P450 family 2 subfamily C member 19 (*CYP2C19*), and cytochrome P450 oxidoreductase (*POR*) to investigate their potential impact on the pharmacokinetics of gefitinib [55, 56, 73]. However, *in vitro* data indicate that CYP1A2, CYP2C9 and CYP2C19 exhibit no measurable metabolic activity towards gefitinib [74]. While some published studies have tried to explore the influence of genetic polymorphisms in these enzymes on gefitinib pharmacokinetics, no functional or kinetic relevance has been demonstrated to date (Table S3).

### Drug transporters

#### Adenosine triphosphate binding cassette subfamily B member 1 (ABCB1)

*ABCB1* is a protein-coding gene with a reverse orientation, composed of 28 exons (transcript: NM_001348946.2 at chromosome 7q21.1: 87,503,017–87,600,884) that codes for a 1280 amino acid long protein, called P-glycoprotein 1 (P-gp). It is an active efflux drug transporter that is involved in the transport of gefitinib after it has been metabolized. Over 79,000 genetic variants have been catalogued in the dbSNP database, with rs1128503, rs2032582, and rs1045642 being the most frequently studied and well-characterized polymorphisms [75, 76].

The SNP, rs1128503 (NM_001348946.2: c.1236 T > C; p. Gly412 =) is a synonymous variant located within exon 12 of the *ABCB1* gene, with C being the minor allele in the Asian population [77]. Several studies have linked this SNP to altered drug exposure, or drug response and even adverse effects of the drugs [78–80]. However, the underlying molecular mechanism as to how this silent SNP is involved in these processes has not been elucidated yet [81]. Moreover, no other studies could infer an association between this polymorphism and gefitinib pharmacokinetics (Table S4).

rs2032582 encodes for a missense mutation (NM_001348946.2: c.2677 T > G/A; p. Ser893Ala/Thr) within the exon 21. Although many studies have examined the association between this nonsynonymous SNP and the phenotypic traits, the results are inconsistent. A study by Ma *et al.,* when investigating this SNP with the treatment outcome of gefitinib has found that patients with GT + TT genotype responded significantly lower than those with GG genotype. The ORR was 51.2% for patients with GT + TT genotypes and 84.6% for patients with GG genotypes (*p* = 0.032) [55]. However, the study could not find any significant correlation between this SNP and the pharmacokinetics of gefitinib. Moreover, other studies have shown that there is no association between this SNP and gefitinib pharmacokinetics (Table S4).

rs1045642, is the most extensively characterized *ABCB1* polymorphism located at exon 26 (NM_001348946.2: c.3435C > T; p.Ile1145 =). Although it leads to a synonymous transition, studies have shown that the TT group is significantly associated with reduced messenger RNA (mRNA) expression and protein stability, which may lead to reduced drug transport activity [82]. However, none of the studies found any association between this SNP and gefitinib pharmacokinetics (Table S4).

#### Adenosine triphosphate binding cassette subfamily G member 2 (ABCG2)

*ABCG2* is a reverse orientation gene that encodes for a 655 amino acid long breast cancer resistance protein (BCRP). *ABCG2* is located on chromosome 4q22.1: 88,090,269–88,158,639, transcript: NM_004827.3 and spans 16 exons. Previous research has found that the BCRP expression and function can be affected by certain naturally occurring variants in *ABCG2* [83]. Studies have investigated the potential association between the SNPs of this gene with the pharmacokinetics of gefitinib (Table 3). The most extensively studied SNP that is linked to gefitinib pharmacokinetics is rs2231142.

**Table 3:** Influence of *ABCG2* genetic variants on PK parameters and efficacy of gefitinib

Authors	Population	Sample size	Criteria for sample collection	Time of sample collection	SNP ID	PK parameters	Efficacy
Li *et al*., 2007 [85]	United States	27 NSCLC patients	Between day 1 to day 28 of 250 mg or 500 mg of gefitinib	Day 1: Predose, 1, 2, 3, 4, 5, 6 and 8 h post-dose Predose samples on Days 2, 3, 8, 15, 22, and 28	rs2231142	Higher gefitinib C_trough_ in patients with C/A genotype than in those with CC	NR
Kobayashi*et al*., 2015 [35]	Japan	31 NSCLC patients	14 days after gefitinib therapy (250 mg OD)	Predose, 1, 2, 4, 6, 8, 12, and 24 h post-dose	rs2231142	AUC_0–24_, C_trough_ – non-significant	NR
Hirose *et al*., 2016 [57]	Japan	35 NSCLC patients	Between day 1 to day 15 of gefitinib therapy (250 mg OD)	Day 1: Predose and 1,3, 5, 8, and 24 h post-dose Day 8: Pre-dose Day 15: Pre-dose	rs2231142	AUC_0–24_, C_max_—non-significant	PFS: non-significant
Ma *et al*., 2019 [55]	Chinese	58 NSCLC patients	Gefitinib therapy—250 mg OD 1 st occasion: Before gefitinib dosage 2nd occasion: Cycle 2^**$**^	0 h (baseline—before first gefitinib dose) and 5 min before gefitinib administration at day 1 of cycle 2^**$**^	rs2231142	Lower gefitinib C_trough_ in patients with AA genotype than in patients with AC + CC	PFS and ORR: non-significant
					rs2231137	C_trough_ – non-significant	PFS and ORR: non-significant
Wan *et al*., 2020 [58]	Chinese	39 healthy volunteers	Single oral dose of 250 mg of gefitinib	Predose and at 0.5, 1, 2, 3, 4, 5, 6, 7, 8, 10, 12, 24, 48, 72, 96, 120, 144 and 168 h post-dose	rs2231142	Higher gefitinib AUC_(0–168 h)_ and AUC_(0-∞)_ in patients with CA + AA genotypes compared to those with CC genotype	NR
					rs2231137	C_max_, T_max_, t_1/2_, AUC_(0–168 h)_, AUC_(0-∞)_, CL/F – non-significant	NR
Sakamoto *et al*., 2020 [84]	Japan	61 NSCLC patients	14 days after gefitinib was administration (250 mg OD)	Predose and at 1, 2, 4, 6, 8, 12, and 24 h post-dose	rs2231142	Lower gefitinib mean C_trough_ and AUC_(0–24)_ in the CA/AA group compared to the CC group	NR
Nio *et al*., 2022 [67]	Japan	18 NSCLC patients who are 75 years or older	Day 1 of gefitinib therapy (250 mg OD)	Predose, 1, 2, 4, 6, 8, 24, and 48 h post-dose	rs2231142	Higher gefitinib AUC_(0–24)_ in patients with the AA genotype than in those with the CC or CA genotype	NR
Chen *et al*., 2024 [53]	Chinese	45 healthy male volunteers	16 days after gefitinib therapy (250 mg OD)	Pre-dose, 0.5, 1, 1.5, 2, 2.5, 3, 3.5, 4, 4.5, 5, 5.5, 6, 6.5, 7, 8, 12, 24, 48, 72, 96, 120 and 144 h post-dose	rs2622604	Shorter gefitinib T_max_ in patients with of CC genotype than in those with CT/TT genotype	NR
					rs7699188	T_max_, t_1/2_, C_max_, AUC_0-t_, AUC_0–1_, Vd, CL/F – non-significant	NR
					rs2231142	T_max_, t_1/2_, C_max_, AUC_0-t_, AUC_0–1_, Vd, CL/F – non-significant	NR
					rs2231137	T_max_, t_1/2_, C_max_, AUC_0-t_, AUC_0–1_, Vd, CL/F – non-significant	NR

^**$**^One month was counted for one cycle*.* Non-significant: refers to instances where the observed changes in PK parameters and efficacy did not reach statistical significance in the cited studies. NR (Not reported): denotes that the cited study did not evaluate or provide data on efficacy. Abbreviations: NSCLC: Non-small cell lung cancer, OD: Once daily, C_trough_: Trough concentration, AUC: Area under the curve, C_max_: Maximum concentration, T_max_: Time of C_max_, t_1/2_: Half-life of drug, Vd: Volume of Distribution, CL/F: Oral plasma clearance, PFS: Progression-free survival, ORR: Objective response rate

rs2231142 (NM_004827.3: c.421C > A; p.Gln141Lys) is a missense variant of *ABCG2* present in exon 5. Ma *et al.,* in their study revealed that patients with the AA genotype exhibited significantly lower median gefitinib C_trough_ (110 ng/mL) compared to those with the AC + CC genotype (200 ng/mL) (*p* = 0.031) [55]. Another study by Wan *et al.,* conducted on healthy volunteers, revealed that AUC_(0–168 h)_ and AUC_(0-∞)_ values of gefitinib were higher by 33% and 37%, respectively, in individuals with CA + AA genotypes than in patients with the CC genotype (*p* < 0.05). Furthermore, they also found that the mean apparent oral clearance (CL/F) was reduced by 32% in patients with the c.421A allele than in those with the CC genotype (37.11 vs 49.83 L/h, *p* = 0.012). Their findings also revealed that the half-life (t_1/2_) value was significantly increased in participants with the A allele (37.51 vs 29.12 h, *p* = 0.038) [58]. On the contrary, results by Sakamoto *et al.,* revealed that patients with CA/AA genotype had a lower mean C_trough_ of gefitinib compared to CC genotype (333.2 ± 188.71 vs 454.5 ± 206.3 ng/mL, respectively, *p* = 0.021), representing approximately 27% lower exposure. Similarly, the AUC_0–24 h_ was significantly reduced in CA/AA carriers (9949.9 ± 5058.94 vs 13,085.4 ± 5075.39 ng.h/mL, *p* = 0.034), corresponding to approximately 24% lower exposure [84]. A study on geriatric NSCLC patients, carried out by Nio *et al.,* reported that those with AA genotype had higher gefitinib AUC_0–48 h_, which was equal to 14.0 ± 1.7 μM h, when compared to patients with CC or CA genotype (AUC_0–48 h_ = 8.58 ± 3.0 μM h) (*p* = 0.0209) [67]. However, a few studies could not establish any association between this SNP and gefitinib pharmacokinetics, as listed in Table 3.

For *ABCG2* rs2231142, reports vary between higher or lower AUC/C_trough_, highlighting potential ethnic differences, methodological variations or confounding factors. Most observed differences range between 30–40%, yet these effects are substantially smaller than gefitinib’s eight to 20-fold overall variability [26]. While mechanistically well-established, rs2231142 does not explain the majority of pharmacokinetic variability, falls within proven safe exposure ranges, and lacks demonstrated predictive value for treatment outcomes, appropriately leading to no recommendations for routine genotyping or dose adjustments in clinical practice guidelines.

#### Solute carrier organic anion transporter family member 1B1 (SLCO1B1)

*SLCO1B1* is a protein-coding gene in the forward strand that comprises 15 exons (transcript: NM_006446.5 at chromosome 12p12.1: 21,131,194–21,239,796). It encodes for an influx transporter protein, an organic anion transporting polypeptide 1B1 (OATP1B1), which is 691 amino acid long and regulates the uptake of substrates from the bloodstream to the hepatocytes. *SLCO1B1* rs2306283 and rs4149056 are the 2 missense polymorphisms that did not affect the gefitinib pharmacokinetics (Table S5) [67].

### Other genes

#### UDP glucuronosyltransferase family 1 member A7 (UGT1A7)

The protein-coding gene, *UGT1A7* present in the forward strand of the deoxyribonucleic acid (DNA) comprises 5 exons (transcript: NM_019077.3 at chromosome 2q37.1: 233,681,901–233,773,300) and encodes for a 530 amino acid long protein that is involved in the phase II biotransformation of multiple drugs. A study by Liu *et al.,* has demonstrated that gefitinib has a competitive inhibition to *UGT1A7* [86]. However, no association was found between the *UGT1A7* variant (rs6759892) and gefitinib pharmacokinetics (Table S5) [55].

## Association of SNPs with gefitinib-induced drug toxicities

As with any medication, gefitinib is also associated with certain ADRs. Unlike chemotherapy and radiation therapy, gefitinib does not cause neutropenia, myelosuppression and alopecia [34, 87]. However, the most common gefitinib-induced ADRs include dermatological (skin rashes, dry skin, acne), gastrointestinal problems (diarrhea, vomiting, nausea) and hepatotoxicity (Fig. 1c) [87, 88]. The most common severe adverse effect is interstitial lung disease (ILD), occurring in approximately 1% of patients globally, with an incidence of 2% reported in Japan [87]. A study has also described gefitinib-related ocular adverse effects [89].

Dermatological ADRs are the most frequently reported, accounting for 71–85% of cases, with mild to moderate skin rashes being the predominant manifestation. In about 18% of patients, these rashes may be severe, requiring dose adjustment or treatment discontinuation [90]. Hepatotoxicity may occur in these patients, with around 5–18% exhibiting more severe forms, specifically grade 3, as defined by the Common Terminology Criteria for Adverse Events (CTCAE) [91, 92]. Diarrhea is a frequently observed gastrointestinal event, occurring in 18–95% of patients in all grades without antidiarrheal prophylaxis [93]. Studies have investigated the relationship between the SNPs within the genes involved in drug metabolism or transport and gefitinib-induced ADRs (Table 4).

**Table 4:** SNPs associated with gefitinib-induced ADRs

Author, Year	Population	Sample size	Gefitinib dosage	Gene/SNP	ADRs
Lemos *et al*., 2011 [94]	Italy	94 NSCLC patients	250 mg OD	*ABCG2* (rs7699188)	Diarrhea
Tamura *et al*., 2012 [83]	Japan	83 NSCLC patients	250 mg OD	*ABCG2* (rs2231137)	Skin rashes
Suzumura *et al*., 2012 [65]	Japan	232 advanced NSCLC patients	Dose not specified	CYP2D6*10 (rs1065852)	Skin rashes
Sugiyama *et al*., 2015 [73]	Japan	60 NSCLC patients	Dose not specified	PM of *CYP2D6* (*5/*10 and *10/*10)	Hepatotoxicity
				*CYP3A5* (rs776746)	Hepatotoxicity
Ma *et al*., 2017 [78]	China	59 NSCLC patients	250 mg OD	*CYP3A4* (rs2242480)	Skin rashes and Diarrhea
				*ABCB1* (rs1128503)	Skin rashes and Diarrhea
				CYP2D6*2 (rs1135840)	Skin rashes
				*CYP3A5* (rs776746)	Diarrhea and Hepatotoxicity
				*POR* (rs1057868, rs17685)	Diarrhea
Guan *et al*., 2021 [95]	China	346 NSCLC patients	250 mg OD	*ABCB1* (rs1128503)	Skin rashes
				*SLC22A1* (rs4709400)	Skin rashes
				*SLC22A8* (rs4149179)	Skin rashes
Kwok *et al*., 2022 [88]	China	152 NSCLC patients	Dose not specified	CYP2D6*41 (rs28371725)	Hepatotoxicity
				CYP2D6*10 (rs1065852)	Skin rashes
				*CYP3A4* (rs2242480)	Diarrhea
Guan *et al*., 2022 [96]	China	180 NSCLC patients	250 mg OD	*FOXO3* (rs4946935)	Hepatotoxicity

NSCLC: Non-small cell lung cancer, OD: Once daily, *ABCG2*: Adenosine triphosphate binding cassette subfamily G member 2, *CYP2D6*: Cytochrome P450 family 2 subfamily D member 6, *CYP3A5*: Cytochrome P450 family 3 subfamily A member 5, *CYP3A4*: Cytochrome P450 family 3 subfamily A member 4, *ABCB1*: Adenosine triphosphate binding cassette subfamily B member 1, *POR*: Cytochrome P450 oxidoreductase, *SLC22A1*: Solute carrier family 22 member 1, *SLC22A8*: Solute carrier family 22 member 8, *FOXO3*: Forkhead box O3

### Dermatological reactions

Suzumura *et al.,* reported a correlation between the CYP2D6*10/*10 diplotype, and a higher incidence of gefitinib-induced severe cutaneous rash (grade 2 or higher) (*p* = 0.03) [65]. Ma *et al.,* found a marginal association between the CYP2D6*2 (rs1135840) and *CYP3A4* rs2242480 with skin rashes (*p* = 0.087 and *p* = 0.068, respectively) [78]. They also revealed a significant association between the TT genotype of *ABCB1* rs1128503 and the likelihood of developing gefitinib-induced skin rashes. Patients with this TT genotype were at a substantially increased risk, experiencing skin rashes 15.78 times more frequently than individuals without this genetic variant. There was also a marginal association between *ABCB1 (*rs1045642) and gefitinib-induced skin rashes (*p* = 0.068) [78]. A study by Kwok *et al.,* performed univariate logistic regression to demonstrate that gefitinib-induced skin rashes were significantly associated with the AA genotype of CYP2D6*10 (rs1065852) (OR (odds ratio) = 3.368; 95% CI (confidence interval) = 1.000–11.345; *p* = 0.050) [88]. A study by Tamura *et al.,* has revealed an association between *ABCG2* rs2231137 and gefitinib-induced skin rashes, where patients with GA or AA genotype had an increased tendency to develop skin rashes than in patients with the homozygous GG genotype (*p* = 0.046) [83]. Genetic variations in other drug transporters such as solute carrier family 22 member 1 (*SLC22A1*) (rs4709400) and solute carrier family 22 member 8 (*SLC22A8*) (rs4149179) are also known to influence gefitinib-induced ADRs. Guan *et al.,* reported that these variants increased the risk of gefitinib-induced skin rashes in NSCLC patients [95].

### Hepatotoxicity

Study findings by Sugiyama *et al.,* revealed that patients with PM phenotype of *CYP2D6* and *CYP3A5* had a significantly higher risk of severe hepatotoxicity compared to those with a non-PM phenotype (*p* = 0.15 and *p* = 0.0123, respectively) [73]. Ma *et al.,* found a strong association between *CYP3A5* rs776746 and hepatotoxicity (*p* = 0.036) [78]. They also revealed an association between rs1057868 and rs17685 of the *POR* gene with gefitinib-induced severe hepatotoxicity (*p* = 0.073 and *p* = 0.0037*,* respectively) [78]. Kwok *et al.,* demonstrated that gefitinib-induced hepatotoxicity was significantly associated with the CT genotype of CYP2D6*41 (rs28371725) (OR = 3.818; 95% CI = 1.062–13.722; *p* = 0.040) [88]. Apart from the genes involved in drug metabolism and transport, a genetic variant in a transcriptional activator, forkhead box O3 (*FOXO3*) has also been investigated for its potential association with the ADRs caused by gefitinib. Guan *et al.,* studied the intronic variant rs4946935 (NM_001455.4: c.*35-288A > G) of *FOXO3* and found a significant association with hepatotoxicity, where the AA carriers had a higher tendency to develop hepatotoxicity than those with GG genotype (*p* = 0.018) [96].

### Gastrointestinal reactions

Ma *et al.,* found a marginal association between *CYP3A4* rs2242480 and severe diarrhea (*p* = 0.083). A strong association between *CYP3A5* rs776746 with severe diarrhea (*p* = 0.021) was also seen [78]. A significant association between *ABCB1* rs1128503 and gefitinib-induced diarrhea was also found, where the patients with TT genotype were at higher risk (10.78 times) for developing diarrhea compared to those without the TT genotype [78]. Kwok *et al.,* demonstrated that gastrointestinal reactions were significantly associated with TT of *CYP3A4* (rs2242480) (OR = 20.000; 95% CI = 2.381–167.965; *p* = 0.006) [88]. Studies have also revealed the association of polymorphisms in the *ABCG2* gene with gefitinib-induced diarrhea [94, 97]. According to a study by Lemos *et al.,* patients with TT genotype in *ABCG2* rs7699188 were significantly more likely to develop grade 2 or 3 diarrhea (*p* < 0.01) [94].

### Interstitial lung disease

In Phase III clinical trials, the incidence of ILD varied across populations, with 1% reported in the Iressa Follow-Up Measure (IFUM) trial among Caucasian patients, and 2.6% in the Iressa Pan-Asia Study (IPASS) trial among Asian patients, suggesting possible ethnic variability in susceptibility [26]. A positive exposure–response (E-R) relationship for ILD was observed in the V-15–33 observational study conducted in Japanese NSCLC patients. The objective of the study was to determine the risk factors of ILD and assess the potential association between exposure and ILD [26]. It was observed that patients in the highest exposure quartile exhibited approximately two-fold higher plasma gefitinib concentrations, and correspondingly greater ILD risk. These findings were supported by multivariate analyses, which maintained the trend after adjusting for other clinical risk factors. Despite these associations, a genome-wide association study (GWAS) conducted using the V-15–33 dataset and a retrospective cohort failed to identify any significant genetic variants associated with ILD risk, underscoring the multifactorial nature of this adverse event [26]. Similar findings were reported by Hirose *et al.,* where there was no significant association found between pharmacokinetics and pharmacogenomics with that of toxicity and efficacy of gefitinib [57]. However, one patient who succumbed to death due to gefitinib-induced ILD, exhibited high AUC and C_max_, along with notable C_trough_, suggesting that elevated systemic exposure to gefitinib may be linked to an increased risk of ILD [57].

## Other factors affecting the pharmacokinetics of gefitinib

### Intrinsic factors

Intrinsic factors refer to patient-specific physiological and genetic characteristics that affect drug absorption, distribution, metabolism, and excretion. Several intrinsic factors can significantly affect the pharmacokinetics of gefitinib, although their clinical significance varies widely. Traditional demographic variables exhibit minimal impact, as population pharmacokinetic analyses consistently indicate that patient age, weight, gender, and ethnicity do not significantly affect gefitinib pharmacokinetics. Caucasian female patients exhibit a higher exposure (∼27%) to gefitinib compared to Caucasian male patients after administration of a single 250 mg oral dose. This increase in exposure is not considered clinically significant, and dose reduction is not required in female patients [26].

Hepatic function significantly influences gefitinib pharmacokinetics. The medication undergoes significant metabolism in the liver, mainly via the cytochrome P450 enzymes, CYP3A4 and CYP2D6. Patients with moderate to severe hepatic impairment due to cirrhosis have almost threefold increased gefitinib exposure compared to those with normal liver function [26]. Despite the significant rise in systemic exposure, no dosage decrease is advised owing to gefitinib's intrinsic high inter-patient pharmacokinetic variability, since exposures across individuals with normal and impaired hepatic function exhibit considerable overlap. The therapeutic justification for maintaining conventional dosing is reinforced by the minimal dose reduction rate of about 10% seen in Phase 2 studies when the gefitinib dosage was increased to 500 mg daily, indicating favorable tolerability even at elevated levels of exposure [26].

### Extrinsic factors

External factors refer to extrinsic effects, including concurrent drugs, dietary habits, and environmental exposures, that may substantially modify drug pharmacokinetics. The most clinically significant extrinsic variables for gefitinib are drug-drug interactions (particularly with CYP-modulating drugs), variations in stomach pH, and diet.

#### Drug-drug interactions with gefitinib

Drug-drug interactions (DDIs) represent a significant and ongoing challenge in the pharmacological management of NSCLC, potentially reducing the therapeutic efficacy and increasing the risk of ADRs [98]. The high prevalence of polypharmacy among NSCLC patients, particularly in advanced stages and the geriatric population, markedly elevates the risk of DDIs [99, 100]. Extensive research has identified clinically relevant DDIs between gefitinib and co-administered medications, necessitating vigilant management strategies [101, 102]. A key mechanism underlying these interactions involves the modulation of CYP450 enzymes, particularly CYP3A4, by concomitant medications, which can function as inducers or inhibitors. Given that gefitinib undergoes extensive metabolism via CYP3A4, the pharmacokinetic profiles are significantly influenced by concurrent administration of CYP3A4 modulators. Potent inducers of CYP3A4 (including dexamethasone, rifampicin, etc.) can accelerate gefitinib metabolism, leading to subtherapeutic drug levels and reduced efficacy, whereas CYP3A4 inhibitors (including itraconazole, efavirenz, aprepitant, fluconazole, etc.) can increase systemic gefitinib exposure, increasing toxicity risks [103–105]. Research has shown that gefitinib can inhibit P-gp activity, thereby resulting in an increased intracellular concentration of the co-administered drugs, potentially leading to DDIs [106]. However, the DDIs affecting gefitinib transport have not been much explored. Given the role of drug transporters in gefitinib pharmacokinetics, further investigations are necessary to determine the extent of which gefitinib may contribute to altered systemic exposure, particularly in combination regimens.

Furthermore, pharmacokinetic interactions extend beyond hepatic metabolism. Gastric pH is a crucial extrinsic factor because of gefitinib's solubility dependence on pH. Gefitinib, a mildly basic compound, has reduced solubility under elevated pH conditions. For instance, proton pump inhibitors (PPIs) such as pantoprazole elevate gastric pH, impairing gefitinib solubility and absorption, reducing its bioavailability and therapeutic potential [102]. Clinical investigations indicate that an increased gastric pH (≥ 5) lowers gefitinib AUC by 47% and C_max_ by 71%. This significant reduction in drug exposure may result in treatment failure. To mitigate this effect, it is recommended that PPIs should not be used concomitantly with gefitinib [107]. In contrast, food intake improves gefitinib absorption. A high-fat meal elevates AUC by 37% and C_max_ by 32% compared to fasting conditions. Nonetheless, this elevation is deemed clinically insignificant, and the medication may be used with or without food. Consistent meal administration may reduce intra-patient variability and enhance pharmacodynamic predictability [26].

An additional layer of complexity arises from phenoconversion, a process in which a patient's metabolic phenotype shifts due to drug-induced enzymatic modulation or genetic predisposition [108]. This phenomenon can alter the expected pharmacokinetics of gefitinib, exacerbating the effects of cytochrome P450 modulation and necessitating individualized therapeutic adjustments [108]. For instance, strong CYP3A4 inhibitors such as itraconazole can increase the AUC of gefitinib by up to 80%, while CYP3A4 inducers like rifampicin can decrease it by a similar magnitude [26]. Similarly, acid-reducing agents such as ranitidine have been shown to reduce gefitinib AUC by 47% due to pH-dependent solubility changes [26].

These large, non-genetic fluctuations in systemic exposure can either mask or exaggerate the effects of genetic polymorphisms, making it challenging to isolate the impact of variants in drug-metabolizing and transporting genes. As such, assessing the clinical relevance of pharmacogenetic findings must be contextualized within this broader variability landscape, including both intrinsic and extrinsic factors. Thus, personalized treatment strategies remain critical in ensuring optimal drug efficacy and minimizing ADRs.

## Limitations and future perspectives

Despite significant advancements in pharmacogenetic research on gefitinib, several limitations persist in the existing literature. This review summarizes the current evidence regarding the impact of genetic polymorphisms on gefitinib pharmacokinetics and adverse effects, while acknowledging the limitations.

Many studies rely on small sample sizes, which may limit the statistical power and the generalizability of findings. Additionally, many of the included studies are based on single SNP assessments, which may not fully capture the complexity of genetic regulation or interactions between genes and the environment. The reproducibility of such findings across different populations and study designs remains a significant concern. Variability in ethnicity, sample size, clinical endpoints, and genotyping methodologies contributes to the lack of consensus across studies.

Furthermore, most SNP associations have not been functionally validated or assessed in prospective clinical trials, limiting their immediate translational potential. Many studies analyze individual SNPs or genotypes in isolation, without considering their combined effects or interactions, which may provide a more accurate representation of pharmacokinetic variability.

Additionally, current safety assessments for genetic variants focus solely on systemic pharmacokinetics, thereby failing to capture the complete safety profile, as local expression of CYPs and transporters in skin and other peripheral organs creates tissue-specific drug concentration and metabolite formation independent of plasma levels [109, 110]. Since transporter expression is highly cell-specific and functional activity varies significantly between tissues [111], relying exclusively on systemic PK parameters may underestimate or overlook tissue-specific risks associated with genetic variants in these genes.

Another key limitation of the pharmacogenetic studies summarized in this review is that most were conducted in patients with advanced NSCLC, where multiple coexisting factors, such as polypharmacy, DDIs, organ impairment, and disease-related physiological changes, can strongly influence gefitinib pharmacokinetics. This contributed to the high background variability in drug exposure, which complicates the interpretation of pharmacogenetic effects. As a result, pharmacogenetic associations may appear inconsistent, sometimes contradictory, or may not be detected at all in certain trials. This inherent variability represents a major limitation of clinical pharmacogenetic investigations of gefitinib and should be considered when evaluating their translational relevance.

Addressing these limitations through larger, multi-ethnic cohort studies and integrative genetic analyses will be essential for improving the clinical utility of pharmacogenetic insights. Future studies should also focus on comprehensive genotyping, including haplotypes or polygenic risk models, functional characterization and validation in diverse, multiethnic cohorts and incorporation of localized gene expression data and tissue drug concentration profiling. Such integrative approaches are essential for enhancing the robustness and clinical applicability of pharmacogenetic insights into gefitinib therapy.

## Conclusion

Pharmacokinetic variability in gefitinib levels poses a significant challenge in the treatment of NSCLC patients. Genetic variants in *ABCB1, ABCG2* and CYP genes may alter the pharmacokinetics of various drugs, including gefitinib, which in turn can influence response to therapy and ADRs. Some literature suggests an association between specific genetic variants of these genes and substantial variations in gefitinib plasma levels, while others report no such association. This suggests the need for more research to better understand these associations and their subsequent clinical implications. To enhance the reliability and translational relevance of such studies, future pharmacogenetic investigations should rigorously account for confounding clinical factors, including organ impairment and concomitant medications. In recent years, genotyping or pharmacogenomics has gained importance due to its promise in individualizing therapy to improve treatment outcomes. Individualized gefitinib dosing, based on pharmacogenetic genotype and proactive monitoring of potential DDIs, with dose modifications, to counteract the impact of CYP3A4 inductions, inhibition, or phenoconversion, ensures improved clinical outcomes for patients with NSCLC.

## Supplementary Information

Below is the link to the electronic supplementary material.

Supplementary file1 (XLSX 17 KB)

Supplementary file2 (DOCX 39 KB)

## Data Availability

No datasets were generated or analysed during the current study.
