# Supplementary material for: Influence of genetic polymorphisms on gefitinib pharmacokinetics and adverse drug reactions in non-small cell lung cancer patients
Source: Cancer Metastasis Rev. 2025 Nov 6;44(4):82. doi: 10.1007/s10555-025-10299-7 (PMC12592279; doi:10.1007/s10555-025-10299-7)
Supplement: Supplementary file 2 — Supplementary file2 (DOCX 39 KB) [file 10555_2025_10299_MOESM2_ESM.docx]

**Table S2:** Influence of rs776746, a *CYP3A5* genetic variant, on PK parameters and efficacy of gefitinib.

| **Authors** | **Population** | **Sample size** | **Criteria for sample collection** | **Time of sample collection** | **PK parameters** | **Efficacy** |
| --- | --- | --- | --- | --- | --- | --- |
| Swaisland *et al*., 2006  (66) | Single centre in the UK and Germany | 73 healthy volunteers | Single oral dose of gefitinib  (50–500 mg) | Scheduled time points for up to 10 days post-dose | CL/F, t_1/2β_ – non-significant | NR |
| Kobayashi, *et al*., 2015  (35) | Japan | 31 NSCLC patients | 14 days after gefitinib therapy   (250 mg OD) | Predose, 1, 2, 4, 6, 8, 12, and 24 h post-dose | AUC_0-24_, C_trough_ –non-significant | NR |
| Hirose *et al*., 2016  (57) | Japan | 35 NSCLC patients | Between day 1 to day 15 of gefitinib therapy  (250 mg OD) | Day 1: Predose and 1,3, 5, 8, and 24 h post-dose Day 8: Pre-dose Day 15: Pre-dose | AUC_0-24_, C_max_ –non-significant | PFS: non-significant |
| Wan *et al*., 2020  (58) | Chinese | 39 healthy volunteers | Single oral dose of 250 mg of gefitinib | Predose and at 0.5, 1, 2, 3, 4, 5, 6, 7, 8, 10, 12, 24, 48, 72, 96, 120, 144 and 168 h post-dose | C_max_, T_max_, t_1/2_, AUC_(0-168h)_, AUC_(0-∞)_, CL/F – non-significant | NR |
| Nio *et al*., 2022  (67) | Japan | 18 NSCLC patients who are 75 years or older | Day 1 of gefitinib therapy (250 mg OD) | Predose, 1, 2, 4, 6, 8, 24, and 48 h post-dose | AUC_0–48_ – non-significant | NR |
| Chen *et al*., 2024  (56) | Chinese | 45 healthy male volunteers | 16 days after gefitinib therapy  (250 mg OD) | 0h (pre-dose), 0.5, 1, 1.5, 2, 2.5, 3, 3.5, 4, 4.5, 5, 5.5, 6, 6.5, 7, 8, 12, 24, 48, 72, 96, 120 and 144h post-dose | Higher gefitinib AUC_0-t_ and AUC_0-∞_  in CC genotype than in CT/TT genotype | NR |

**Footnote:** Non-significant: refers to instances where the observed changes in PK parameters and efficacy did not reach statistical significance in the cited studies. NR (Not reported): denotes that the cited study did not evaluate or provide data on efficacy. Abbreviations: NSCLC: Non-small cell lung cancer, OD: Once daily, AUC: Area under the curve, C_trough_: Trough concentration, C_max_: Maximum concentration, T_max_: Time of C_max_, t_1/2_: Half-life of drug, t_1/2β_ = terminal elimination half-life, CL/F: Oral plasma clearance, PFS: Progression-free survival

**Table S3:** Influence of other CYP genetic variants on PK parameters and efficacy of gefitinib.

| **Authors** | **Population** | **Sample size** | **Criteria for sample collection** | **Time of sample collection** | **Gene and SNP ID** | **PK parameters** | **Efficacy** |
| --- | --- | --- | --- | --- | --- | --- | --- |
| Ma *et al*., 2019  (55) | Chinese | 58 NSCLC patients | Gefitinib therapy – 250 mg OD  1^st^ occasion: Before gefitinib dosage  2^nd^ occasion: Cycle 2**^$^** | 0h (baseline - before first gefitinib dose) and 5 minutes before gefitinib administration on day 1 of cycle 2**^$^** | *CYP1A1* - rs2606345 | C_trough_ – non-significant | PFS and ORR: non-significant |
|  |  |  |  |  | *CYP1A1* - rs1048943 | C_trough_ – non-significant | PFS and ORR: non-significant |
|  |  |  |  |  | *CYP1A2* - rs762551 | C_trough_ – non-significant | PFS and ORR: non-significant |
|  |  |  |  |  | *POR* - rs1057868 | C_trough_ – non-significant | PFS and ORR: non-significant |
|  |  |  |  |  | *POR* - rs17685 | C_trough_ – non-significant | PFS and ORR: non-significant |
| Wan *et al*., 2020  (58) | Chinese | 39 healthy volunteers | Single oral dose of 250 mg of gefitinib | Predose and at 0.5, 1, 2, 3, 4, 5, 6, 7, 8, 10, 12, 24, 48, 72, 96, 120, 144, and 168 h post-dose | *CYP2C9*3* - rs1057910 | C_max_, T_max_, t_1/2_, AUC_(0-168h)_, AUC_(0-∞)_, CL/F – non-significant | NR |
|  |  |  |  |  | *CYP2C19*2* - rs4244285 | C_max_, T_max_, t_1/2_, AUC_(0-168h)_, AUC_(0-∞)_, CL/F – non-significant | NR |
|  |  |  |  |  | *CYP2C19*3* - rs4986893 | C_max_, T_max_, t_1/2_, AUC_(0-168h)_, AUC_(0-∞)_, CL/F – non-significant | NR |
| Chen *et al*., 2024  (56) | Chinese | 45 healthy male volunteers | 16 days after gefitinib therapy  (250 mg OD) | Pre-dose, 0.5, 1, 1.5, 2, 2.5, 3, 3.5, 4, 4.5, 5, 5.5, 6, 6.5, 7, 8, 12, 24, 48, 72, 96, 120 and 144h post-dose | *CYP2C9*3* - rs1057910 | Higher gefitinib AUC_0-t_, AUC_0-∞_ and C_max_ in the carrier of AC genotype than in AA genotype | NR |
|  |  |  |  |  | *CYP2C19*2* - rs4244285 | T_max_, t_1/2_ , C_max_, AUC_0-t_ ,  AUC_0-1_, Vd,  CL/F – non-significant | NR |
|  |  |  |  |  | *CYP2C19*3* - rs4986893 | T_max_, t_1/2_ , C_max_, AUC_0-t_ ,  AUC_0-1_ , Vd,  CL/F – non-significant | NR |

**Footnote: ^$^**One month was counted for one cycle. Non-significant: refers to instances where the observed changes in PK parameters and efficacy did not reach statistical significance in the cited studies. NR (Not reported): denotes that the cited study did not evaluate or provide data on efficacy. Abbreviations: NSCLC: Non-small cell lung cancer, OD: Once daily, *CYP1A1*: Cytochrome P450 family 1 subfamily A member 1, *CYP1A2*: Cytochrome P450 family 1 subfamily A member 2, *POR*: Cytochrome P450 oxidoreductase, *CYP2C9*: Cytochrome P450 family 2 subfamily C member 9, *CYP2C19*: Cytochrome P450 family 2 subfamily C member 19, C_trough_: Trough concentration, AUC: Area under the curve, C_max_: Maximum concentration, T_max_: Time of C_max_, t_1/2_: Half-life of drug, Vd: Volume of Distribution, CL/F: Oral plasma clearance, ORR: Objective response rate, PFS: Progression-free survival

**Table S4:** Influence of *ABCB1* genetic variants on PK parameters and efficacy of gefitinib.

| **Authors** | **Population** | **Sample size** | **Criteria for sample collection** | **Time of sample collection** | **SNP ID** | **PK parameters** | **Efficacy** |
| --- | --- | --- | --- | --- | --- | --- | --- |
| Li *et al*., 2007  (85) | United States | 27 NSCLC patients | Between day 1 to day 28 of 250 mg or 500 mg of gefitinib | Day 1: Predose, 1, 2, 3, 4, 5, 6 and 8 h post-dose  Day 2, 3, 8, 15, 22, and 28: Predose | rs1045642 | C_trough_ – non-significant | NR |
| Kobayashi *et al*., 2015  (35) | Japan | 31 NSCLC patients | 14 days after gefitinib therapy   (250 mg OD) | Predose, 1, 2, 4, 6, 8, 12, and 24 h post-dose | rs1128503 | AUC_0-24_, C_trough_ – non-significant | NR |
|  |  |  |  |  | rs2032582 | AUC_0-24_, C_trough_ – non-significant | NR |
|  |  |  |  |  | rs1045642 | AUC_0-24_, C_trough_ – non-significant | NR |
| Hirose *et al*., 2016  (57) | Japan | 35 NSCLC patients | Between day 1 to day 15 of gefitinib therapy  (250 mg OD) | Day 1: Predose and 1,3, 5, 8, and 24 h post-dose Day 8: Pre-dose Day 15: Pre-dose | rs1128503 | AUC_0-24_, C_max_ - non-significant | PFS: non-significant |
|  |  |  |  |  | rs2032582 | AUC_0-24_, C_max_ - non-significant | PFS: non-significant |
|  |  |  |  |  | rs1045642 | AUC_0-24_, C_max_ - non-significant | PFS: non-significant |
| Ma *et al*., 2019  (55) | Chinese | 58 NSCLC patients | Gefitinib therapy – 250 mg OD  1^st^ occasion: Before gefitinib dosage  2^nd^ occasion: Cycle 2**^$^** | 0h (baseline - before first gefitinib dose) and 5 minutes before gefitinib administration at day 1 of cycle 2**^$^** | rs1128503 | C_trough_ – non-significant | PFS and ORR: non-significant |
|  |  |  |  |  | rs2032582 | C_trough_ – non-significant | ORR was better in patients with  GG phenotype than patients with GT + TT phenotypes (84.6% *vs.* 51.2%, P=0.032) |
|  |  |  |  |  | rs10256836 | C_trough_ – non-significant | Patients with GG phenotype achieved longer PFS than patients with GC + CC phenotypes (17.40 *vs.* 10.33 months, P=0.040) |
|  |  |  |  |  | rs1045642 | C_trough_ – non-significant | PFS and ORR: non-significant |
| Wan *et al*., 2020  (58) | Chinese | 39 healthy volunteers | Single oral dose of 250 mg of gefitinib | Predose and at 0.5, 1, 2, 3, 4, 5, 6, 7, 8, 10, 12, 24, 48, 72, 96, 120, 144 and 168 h post-dose | rs1128503 | C_max_, T_max_, t_1/2_, AUC_(0-168h)_, AUC_(0-∞)_, CL/F – non-significant | NR |
|  |  |  |  |  | rs2032582 | C_max_, T_max_, t_1/2_, AUC_(0-168h)_, AUC_(0-∞)_, CL/F – non-significant | NR |
|  |  |  |  |  | rs1045642 | C_max_, T_max_, t_1/2_, AUC_(0-168h)_, AUC_(0-∞)_, CL/F – non-significant | NR |
| Nio *et al*., 2022  (67) | Japan | 18 NSCLC patients who are 75 years or older | Day 1 of gefitinib therapy (250 mg OD) | Predose, 1, 2, 4, 6, 8, 24, and 48 h post-dose | rs1128503 | AUC_0–48_ – non-significant | NR |
|  |  |  |  |  | rs2032582 | AUC_0–48_ – non-significant | NR |
|  |  |  |  |  | rs1045642 | AUC_0–48_ – non-significant | NR |
| Chen *et al*., 2024  (56) | Chinese | 45 healthy male volunteers | 16 days after gefitinib therapy  (250 mg OD) | Pre-dose, 0.5, 1, 1.5, 2, 2.5, 3, 3.5, 4, 4.5, 5, 5.5, 6, 6.5, 7, 8, 12, 24, 48, 72, 96, 120 and 144h post-dose | rs1045642 | T_max_, t_1/2_, C_max_, AUC_0-t_ ,  AUC_0-1_, Vd,  CL/F – non-significant | NR |

**Footnote: ^$^**One month was counted for one cycle. Non-significant: refers to instances where the observed changes in PK parameters and efficacy did not reach statistical significance in the cited studies. NR (Not reported): denotes that the cited study did not evaluate or provide data on efficacy. Abbreviation: NSCLC: Non-small cell lung cancer, OD: Once daily, C_trough_: Trough concentration, AUC: Area under the curve, C_max_: Maximum concentration, T_max_: Time of C_max_, t_1/2_: Half-life of drug, Vd: Volume of Distribution, CL/F: Oral plasma clearance, ORR: Objective response rate, PFS: Progression-free survival

**Table S5:** Influence of genetic variants of other genes on PK parameters and efficacy of gefitinib.

| **Authors** | **Population** | **Sample size** | **Criteria for sample collection** | **Time of sample collection** | **Gene and SNP ID** | **PK parameters** | **Efficacy** |
| --- | --- | --- | --- | --- | --- | --- | --- |
| Ma *et al*., 2019  (55) | Chinese | 58 NSCLC patients | Gefitinib therapy – 250 mg OD  1^st^ occasion: Before gefitinib dosage  2^nd^ occasion: Cycle 2**^$^** | 0h (baseline - before first gefitinib dose) and 5 minutes before gefitinib administration on day 1 of cycle 2**^$^** | *UGT1A7*: rs6759892 | C_trough_ – non-significant | PFS and ORR: non-significant |
| Nio *et al*., 2022  (67) | Japan | 18 NSCLC patients who are 75 years or older | Day 1 of gefitinib therapy (250 mg OD) | Predose, 1, 2, 4, 6, 8, 24, and 48 h post-dose | *OATP1B1*: rs2306283 | AUC_0–48_ – non-significant | NR |
|  |  |  |  |  | *OATP1B1*: rs4149056 | AUC_0–48_ – non-significant | NR |

**Footnote: ^$^**One month was counted for one cycle. Non-significant: refers to instances where the observed changes in PK parameters and efficacy did not reach statistical significance in the cited studies. NR (Not reported): denotes that the cited study did not evaluate or provide data on efficacy. Abbreviations: NSCLC: Non-small cell lung cancer, OD: Once daily, *UGT1A7*: UDP glucuronosyltransferase family 1 member A7, *OATP1B1*: organic anion transporting polypeptide 1B1, C_trough_: Trough concentration, AUC: Area under the curve, ORR: Objective response rate, PFS: Progression-free survival
